# Androgenic Alopecia Is Associated with Less Dietary Soy, Higher Blood Vanadium and rs1160312 1 Polymorphism in Taiwanese Communities

**DOI:** 10.1371/journal.pone.0079789

**Published:** 2013-12-30

**Authors:** Ching-Huang Lai, Nain-Feng Chu, Chi-Wen Chang, Shu-Li Wang, Hsin-Chou Yang, Chi-Ming Chu, Chu-Ting Chang, Ming-Huang Lin, Wu-Chien Chien, Sui-Lung Su, Yu-Ching Chou, Kang-Hua Chen, Wei-Ming Wang, Saou-Hsing Liou

**Affiliations:** 1 Department of Epidemiology, School of Public Health, National Defense Medical Center, Taipei, Taiwan; 2 Taitung Hospital, Department of Health, Executive Yuan, Taiwan; 3 School of Nursing, College of Medicine, Chang-Gung University, Taoyuan, Taiwan; 4 Division of Environmental Health and Occupational Medicine, National Health Research Institutes, MiaoLi, Taiwan; 5 Institute of Statistical Science Academia Sinica, Taipei, Taiwan; 6 Department of Dermatology, Tri-Service General Hospital, Taipei, Taiwan; The Ohio State University, United States of America

## Abstract

**Background:**

Although the genetic basis of androgenic alopecia has been clearly established, little is known about its non-genetic causes, such as environmental and lifestyle factors.

**Objective:**

This study investigated blood and urine heavy metals concentrations, environmental exposure factors, personal behaviors, dietary intakes and the genotypes of related susceptibility genes in patients with androgenic alopecia (AGA).

**Design:**

Age, AGA level, residence area, work hours, sleep patterns, cigarette usage, alcohol consumption, betel nut usage, hair treatments, eating habits, body heavy metals concentrations and rs1998076, rs913063, rs1160312 and rs201571 SNP genotype data were collected from 354 men. Logistic regression analysis was performed to examine whether any of the factors displayed odds ratios (ORs) indicating association with moderate to severe AGA (≧IV). Subsequently, Hosmer-Lemeshow, Nagelkerke R^2^ and accuracy tests were conducted to help establish an optimal model.

**Results:**

Moderate to severe AGA was associated with the AA genotype of rs1160312 (22.50, 95% CI 3.99–126.83), blood vanadium concentration (0.02, 95% CI 0.01–0.04), and regular consumption of soy bean drinks (0.23, 95% CI 0.06–0.85), after adjustment for age. The results were corroborated by the Hosmer-Lemeshow test (P = 0.73), Nagelkerke R^2^ (0.59), accuracy test (0.816) and area under the curve (AUC; 0.90, 0.847–0.951) analysis.

**Conclusions:**

Blood vanadium and frequent soy bean drink consumption may provide protect effects against AGA. Accordingly, blood vanadium concentrations, the AA genotype of rs1160312 and frequent consumption of soy bean drinks are associated with AGA.

## Introduction

The incidence of androgenic alopecia (AGA) is increasing, while the age of onset of AGA continues to decrease. Studies have associated AGA with a variety of diseases, such as coronary heart disease [1], [2], [3], hypertension [4], prostate cancer [5], [6], and ischemic heart disease [7], and it is likely that AGA is a precursor symptom of these diseases. In 2007, Merck & Co. reported US$405 million in global sales of medical products related to AGA treatment, highlighting the tremendous social and economic impact of AGA [8],[9]. Moreover, AGA has important effects on mental health due to the changes in physical appearance that are caused by hair loss.

Many studies have been devoted to the genetic and androgen-related aspects of AGA [8], [10], [11],[12]. Based on a screening of 1025 blood samples from men aged 35 to 75, Richards *et al.* identified a baldness susceptibility gene that increases the risk of AGA six-fold; the variance explained by this allele was reported to be 13.7% [8]. Nyholt *et al.* [13] reasoned that the major contributing factor to AGA is heredity, which accounts for 80% of the variance. However, the genetic aspect lacks specificity because an individual carrying a risk-associated allele will not suffer from AGA until he or she reaches a certain age. This indication that AGA risk alleles are modulated by age is consistent with the world-wide increase in the prevalence of AGA with age. Consequently, earlier onset AGA is associated with more severe characteristics [14].

To date, three AGA susceptibility genes have been identified: the *AR* gene on the X chromosome and two autosomal loci, 3q26 [15] and 20p11 [8], [10]. Richards *et al.* observed that variants in the 20p11 locus and the *AR* gene are common among Europeans and that men with at least one risk allele (20p11.22 or *AR*) at either locus have a seven-fold greater probability of developing AGA than those without either risk allele [8]; carriers of at least one risk allele accounted for one-seventh of all men in the study. Hillmer *et al.* also showed that the 20p11 locus is associated with early-onset AGA [10], [11].

In addition, Hillmer *et al.* discovered that DNA short tandem repeats on chromosome 3 (namely D3S3053, D3S1556 and D3S2425) are related to AGA [15]. Chen *et al* [16] reported that the expression level of *SRY* increases with the severity of baldness. Therefore, we attempted to investigate two single nucleotide polymorphisms (SNPs) within the *SRY* gene.

Although a strong genetic basis for AGA has been established, little is known about its non-genetic causes, such as environmental and dietary factors. This study investigated the bodily heavy metals concentrations, dietary habits and genotypes of related susceptibility genes in patients with AGA.

It has been suggested that air pollution may lead to the over-accumulation of certain heavy metals in the scalp, resulting in hair loss [17],[18]. A study conducted in Lithuania reported that bald individuals had higher concentrations of lead, copper and cadmium and lower concentrations of zinc in their hair follicles than did individuals with normal hair [18]. It has been proposed that lead may replace zinc in heme, while cadmium substitutes for zinc in metallothionein, and the combination of these losses of zinc likely cause alopecia [19].

Smoking also affects the development of AGA because the genotoxic compounds in cigarettes may damage the DNA in hair follicles and subsequently cause microvascular poisoning in hair papillae [20]. Studies have established that a family history of AGA, the age of AGA onset (age ≤40 years old) and smoking are all correlated with AGA [9].

Despite the discovery of genes associated with this disorder, many factors contributing to the variable levels of AGA have yet to be elucidated. To date, no study has comprehensively examined many of the potential AGA-associated factors, such as dietary and body concentrations of heavy metals. Thus, we attempted to assess the association between the physiological concentrations of heavy metals, dietary factors and susceptibility genes in men with AGA.

## Materials and Methods

### Research subjects

The research subjects were men from 35 to 65 years of age who had lived in the districts of Dadu, Longjing and Shigang in Taichung for at least 5 consecutive years. Age and relocation time were used to divide individuals who met these criteria into three age groups (35–44, 45–54 and 55–64). Subsequently, 13 villages were selected as research sites based on their registered permanent residents; each village contained 40 individuals in each of the three age groups. A total of 1560 men were examined.

Consent letters and survey forms were mailed to the subjects from the sample list, and appointments were arranged at local health centers. The signed consent letters were collected at these appointments. A total of 354 men agreed to participate in the study. A physical examination, specimen collection and photographic documentation of the scalp were performed by nurses in these public health centers.

A matched case-control study was conducted with controls recruited from the communities in the previously defined districts of Dadu, Longjing and Shigang in Taichung of central Taiwan. Eligibility was restricted to locally registered residents who had lived in the selected areas for more than five consecutive years and who were between 35 and 64 years old.

Subjects were recruited from five villages within the Longjing and Dadu townships. The reference group was recruited from the two villages of the Shihgang Township, an area located in northeast Taichung County that is free of industrial pollution. Eligible subjects were registered local residents who had lived in the selected areas for more than five consecutive years and who were between 35 and 64 years old. A total of 1440 subjects were randomly selected from each age group (35, 45, and 55 years old), gender (male and female) and village, i.e., a total of 12 subgroups. This study focused on AGA in men. A total of 354 men agreed to participate in the study, for a response rate of 49.2% (354/720); 277 of the subjects were from the exposed area, and 77 were from the reference area.

This study was approved by the institutional review board at the Tri-Service General Hospital in Taipei, Taiwan. Study consent letters and questionnaires were mailed to the subjects from the sample list, and appointments were arranged at local health centers. All the participants signed informed consent waivers prior to study enrollment. The examination, specimen collection and photographic documentation of the scalp were performed at the public health centers.

### Data collection

#### Demographic and medical data

At the beginning of the study, we mailed a self-administered questionnaire to the participants. The questionnaire gathered information about personal characteristics and lifestyle information, such as age, education, smoking habits, alcohol consumption, betel nut usage, work hours, sleep patterns, hair treatments, disease history, and eating habits. Subjects were asked to collect a first void urine sample using a 100 ml polyethylene container on the morning of their appointment. The subjects brought their survey questionnaires and urine samples to the public health centers. The intra-class correlation of dietary intake from 65 studies was 0.9 for two food frequency questionnaires (FFQ) administered 1 month apart, with correlations of 0.4 to 0.8 for various nutrients [21]. The urine samples were labeled with the subject identification number, date, and time. The samples were transported in a cooler and stored at 4°C during shipping. Aliquots were prepared and stored in a −80°C freezer.

The blood samples were collected by venipuncture from the forearm in either (1) trace element k_2_ EDTA tubes (Becton Dickinson, Rutherford, New Jersey) to evaluate heavy metal concentrations or (2) EDTA tubes (Becton Dickinson, Rutherford, New Jersey) to extract DNA. The blood collection tubes were transported in a cooler and stored at 4°C during shipping to the laboratory. The blood samples were divided into aliquots for heavy metal analyses. For the DNA extraction, the EDTA-containing whole blood was centrifuged for 15 min, and the resulting buffy coat containing the white blood cells was subjected to genomic DNA extraction. SNP genotyping was performed using the ABI TaqMan® system. The degree of AGA was evaluated based on the Hamilton-Norwood scale by specialists at the public health centers.

### Detection of susceptibility genes from blood samples

The keyword “androgenic alopecia” was used to search the Online Mendelian Inheritance in Man (OMIM) database for human studies, yielding three accession numbers: 109200, 300710 and 612421. These accession numbers were used in a literature search, which identified AGA-associated genes located on chromosomes X [11], [22], [23], 3 [15] and 20 [8], [10] (Table S1 in File SI). We selected the following 11 most relevant SNPs (Table S2 and S3 in File SI; criteria listed below): rs925391 (the polymorphism with the highest odds ratio), rs10521339 (the polymorphism with the lowest P value), rs6152 (a polymorphism reported independently by Hillmer *et al.* and Ellis *et al.*) and rs6625163 (the most recently identified locus, identified on the X chromosome by Richards *et al.* via a genome-wide screen) located within Xq12–Xq13; rs1158928 (the highest odds ratio) located within Xq11–Xq12; rs1998076 (the polymorphism with the highest odds ratio in the study by Hillmer *et al.*), rs1160312 (the polymorphism with the lowest P value reported by Richards *et al.*), rs201571 (the polymorphism reported by Hillmer *et al.* as associated with AGA and reported by Richards *et al.* to possess linkage disequilibrium with the rs1160312 locus) and rs913063 (used to verify an r^2^ of 1 between this locus and rs1160312) located within 20p11.1–20p11.2. No polymorphisms in rs925391, rs10521339, rs6625163, rs1158928, rs6152 or rs2534636 were found among Asians. However, five of the variants, rs1998076, rs913063, rs1160312, rs201571 and rs11575897, were found to be polymorphic among Asians. Thus, the latter five SNPs were used for the genotyping tests.

### Measurements of the concentrations of heavy metals in the blood and urine

Inductively coupled plasma mass spectrometry (ICP-MS) (Agilent 7700× series ICP-MS, Agilent Technologies, Inc., Palo Alto, CA, USA) was used to measure the concentrations of heavy metals (including vanadium, manganese, cobalt, copper, zinc, arsenic, cadmium, lead, nickel, and chromium) in the blood and urine samples.

The recovery efficiencies from urine samples were determined by spiking a known quantity of a trace element (NIST SRM ®2670a) into a urine sample and following the same experimental procedure used for the treatment of urine samples. The recoveries were as follows: V, 102%; Mn, 103%; Co, 104%; Cu, 102%; Zn, 94%; As, 104%; Cd, 104%; Pb, 104%; Ni, 105%; and Cr, 99%. The blank tests for metals were performed using the same procedure used in the recovery efficiency tests but without adding the known standard solution. The limits of detection were as follows (in µg/L): V, 0.028; Mn, 0.027; Co, 0.004; Cu, 0.025; Zn, 0.075; As, 0.027; Cd, 0.017; Pb, 0.026; Ni, 0.032; and Cr, 0.037. At these limits, the signal-to-noise ratio was 3.

The recovery efficiencies from blood samples were determined by spiking a known quantity of trace elements (Seronorm™) into a blood sample and following the same experimental procedure used for the treatment of the blood samples. The recoveries were as follows: V, 104%; Mn, 101%; Co, 97%; Cu, 95%; Zn, 103%; As, 96%; Cd, 98%; Pb, 100%; Ni, 97%; and Cr, 104%. The limits of detection were as follows (in µg/L): V, 0.051; Mn, 0.018; Co, 0.006; Cu, 0.047; Zn, 0.032; As, 0.039; Cd, 0.013; Pb, 0.012; Ni, 0.019; and Cr, 0.032. At these limits, the signal-to-noise ratio was 3.

The analyses of the blanks, including field blanks and lab blanks, revealed no significant contamination (i.e., the ICP-MS integrated area was below the detection limit). All sample preparation and measurement steps were performed in a laminar flow cabinet.

### Statistical analysis

Statistical analyses were performed using SPSS 17.0. Continuous variables are expressed as the means with standard errors. Categorical variables are expressed as percentages. The Bonferroni adjustment for multiple testing was performed using SISA [24] to control for a family-wise error rate of 0.05, which significant level is considered as 0.05/42 = 0.00114. The p-values in the tables are reported in scientific notation if too many digits were needed for evaluation and to address the issue of multiple testing.

The gene-counting method was used to estimate the genotype frequencies of individual SNPs, which employs the standard EM algorithm for haplotype frequency estimation [25]. Stata 8 and its associated macro programs were used to examine whether a SNP was in Hardy-Weinberg equilibrium [24], [26]. Univariate logistic regression analysis was performed to analyze whether an allele of a SNP had an odds ratio indicating association with moderate to severe AGA (≧IV); subsequently, multivariable logistic regression was used to adjust the relevant confounding factors. In addition, the Hosmer-Lemeshow, explained variance (Nagelkerke R^2^) and accuracy tests were employed to help establish an optimal model.

## Results

Our study population included 60 men with moderate to severe AGA (≥IV). A total of 17.6% of the men aged 46–55 and 27% of men over 55 displayed moderate to severe AGA. These data indicate that the prevalence of AGA in men increased with age (Table 1). Additionally, our data indicated that for each increase of 1 year in age, the odds ratio of moderate to severe AGA was 1.08 (95% CI 1.04–1.13). The odds ratio of moderate to severe AGA for a positive family history was 8.57 (95% CI 3.35–25.77). Age and family history remained significant factors for moderate to severe AGA in our multivariate analysis.

**Table 1:** Male androgenic alopecia frequency by age group.

Age group n(%)				
Type	35–45	46–55	56–65	Total
I	66(52.8)	50(42.0)	29(26.4)	145
II	26(20.8)	26(21.8)	20(26.4)	72
IIa*	2(1.6)	3(2.5)	3(2.7)	8
III	13(10.4)	13(10.9)	16(14.5)	42
IIIa	0(0)	1(0.8)	1(0.9)	2
IIIv	9(7.2)	5(4.2)	11(10.0)	25
Subtotal III to IIIv	22(17.6)	19(15.9)	28(25.4)	69
IV	3(2.4)	7(5.9)	6(5.5)	16
IVa	1(0.8)	2(1.7)	0(0)	3
Subtotal IV to IVa	4(3.2)	9(2.6)	6(5.5)	19
V	4(3.2)	2(1.7)	4(3.6)	10
Va	0(0)	2(1.7)	4(3.6)	6
Subtotal V	4(3.2)	4(3.4)	8(7.2)	16
VI	1(0.8)	3(2.5)	7(6.4)	11
VII	0(0)	5(4.2)	9(8.2)	14
Subtotal ≧IV	9(7.2)	21(12.7)	30(27.3)	60
Total	125	119	110	354

### Body concentrations of heavy metals and AGA

The geometric mean concentrations (µg/L) of ten heavy metals in the blood samples of participants with and without AGA are shown in Table 2. These concentration differences were statistically significant based on the univariate analysis.

**Table 2:** Simple and general linear regression of heavy metal concentrations in male subjects with androgenic alopecia.

Variables			Univariate Analysis		Multivariate Analysis^a^		Multivariate Analysis^b^	
	Type I–III	Type IV–VII	Odds ratio		Adjusted odds ratio		Adjusted odds ratio	
Unit: µg/L	Mean (SD)	Mean (SD)	(95% CI)	P value	(95% CI)	P value	(95% CI)	P value
Blood								
V	117.49 (1.70)	93.33 (1.62)	0.16 (0.05–0.56)	3.87E-3	0.02 (0.00–0.16)	1.36E-4*	0.001 (0.00–0.71)	0.04
Mn	21.38 (2.29)	16.22 (2.63)	0.41 (0.19–0.87)	0.02	0.19 (0.07–0.55)	1.97E-3	9.33 (0.62–139.62)	0.11
Co	0.83 (1.78)	0.66 (2.00)	0.20 (0.07–0.61)	4.64E-3	0.05 (0.01–0.25)	2.58E-4*	0.02 (0.00–1.78)	0.09
Cu	794.33 (1.95)	549.54 (2.19)	0.22 (0.10–0.52)	4.60E-4*	0.08 (0.02–0.28)	9.41E-5*	0.01 (0.00–2.15)	0.10
Zn	4265.80 (1.95)	3019.95 (1.95)	0.18 (0.07–0.45)	2.98E-4*	0.08 (0.02–0.29)	1.86E-4*	0.75 (0.01–77.96)	0.90
As	46.77 (1.74)	36.31 (1.66)	0.14 (0.04–0.48)	1.87E-3	0.02 (0.00–0.16)	1.45E-4*	90.65 (0.10–86871.09)	0.20
Cd	9.55 (2.34)	0.14 (2.09)	0.27 (0.18–0.57)	6.25E-4*	0.18 (0.06–0.53)	1.71E-3	0.83 (0.17–4.12)	0.82
Pb	38.90 (2.19)	29.51 (2.24)	0.35 (0.16–0.77)	0.01	0.19 (0.06–0.57)	3.31E-3	0.66 (0.08–5.30)	0.70
Ni	12.02 (2.09)	9.33 (2.57)	0.42 (0.20–0.88)	0.02	0.26 (0.09–0.76)	0.01	5.15 (0.39–67.99)	0.21
Cr	416.87 (2.00)	309.03 (2.19)	0.30 (0.13–0.67)	3.32E-3	0.14 (0.04–0.45)	1.08E-3*	41.55 (0.45–3832.25)	0.11
Urine								
V	45.71 (1.74)	44.67 (1.62)	0.77 (0.24–2.52)	0.67	1.24 (0.26–5.98)	0.79	3.68 (0.11–121.99)	0.47
Mn	1.07 (4.37)	0.85 (5.89)	0.81 (0.54–1.21)	0.31	0.80 (0.47–1.35)	0.40	0.66 (0.37–1.18)	0.16
Co	0.76 (2.00)	0.76 (2.34)	1.08 (0.44–2.64)	0.87	1.60 (0.44–5.83)	0.48	3.38 (0.17–67.20)	0.43
Cu	47.86 (2.34)	47.86 (2.63)	0.96 (0.47–1.98)	0.92	1.48 (0.53–4.10)	0.46	1.65 (0.39–6.96)	0.50
Zn	562.34 (1.95)	616.60 (1.86)	1.51 (0.56–4.06)	0.42	1.80 (0.47–6.86)	0.39	2.4 (0.37–15.62)	0.36
As	120.23 (2.51)	117.49 (3.39)	0.97 (0.50–1.87)	0.92	1.02 (0.44–2.35)	0.96	0.91 (0.27–3.03)	0.88
Cd	0.74 (3.09)	0.81 (2.40)	1.19 (0.65–2.19)	0.57	0.94 (0.39–2.25)	0.89	0.46 (0.15–1.44)	0.18
Pb	0.83 (6.46)	1.35 (6.31)	1.42 (0.97–2.07)	0.68	1.44 (0.86–2.41)	0.17	1.41 (0.81–2.47)	0.23
Ni	12.02 (2.57)	12.59 (2.75)	1.10 (0.56–2.19)	0.78	1.25 (0.44–3.52)	0.67	0.64 (0.07–5.53)	0.68
Cr	141.25 (1.74)	131.83 (1.70)	0.62 (0.20–1.92)	0.41	1.11 (0.25–4.81)	0.89	0.25 (0.01–8.28)	0.44

Statistical significance with Bonferroni Correction (P<1.14E-3).

^a^ Adjusted for age and family history of AGA.

^b^ Adjusted for age, family history of AGA and the concentrations of other heavy metals.

In the multivariate analysis, after adjusting for age and family history, the odds ratios for the body concentrations of all tested heavy metals and between the two groups (with and without moderate to severe AGA) were statistically significant (P<0.00119). In addition, the correlation coefficients between the body concentrations of all ten metals were statistically significant. Therefore, the concentrations of heavy metals were subjected to further mutual adjustment, which showed that vanadium was the sole element with an odds ratio that reached 0.001 for moderate to severe AGA (P = 0.04).

The geometric mean urine concentrations (µg/L) of the heavy metals in the two groups (with and without AGA) are shown in Table 2. None of the concentration differences were statistically significant based on the univariate analysis. In the multivariate analysis, after adjustment for age and family history, none of the urine metals had an odds ratio indicating a statistically significant association with moderate to severe AGA. Likewise, after adjustment for age, family history and the concentrations of the ten metals, no other factor was found to have a statistically significant odds ratio for association with AGA.

### Environmental, behaviors, dietary factors and moderate to severe AGA

The relationship between environmental factors, personal behaviors, dietary factors and moderate to severe AGA is illustrated in Table 3. In the univariate analysis, every increase of 1 hour of sleep generated an odds ratio of 0.69 for moderate to severe AGA (P<0.01). In contrast, poor sleep quality (inadequate sleep time, difficulty falling asleep or sleep interruption) and scalp abnormalities (itchy, oily or dry) had odds ratios of 1.96 (P = 0.02) and 2.51 (P<0.01), respectively, for the development of moderate to severe AGA. In addition, compared with subjects who rarely drink soy bean products, regular soy bean drinkers (at least 1–3 days per week) had an odds ratio of 0.04 (P = 0.04) for developing moderate to severe AGA.

**Table 3:** Simple and general linear regression analyses of male androgenic alopecia subjects associated with environmental factors, personal behaviors and dietary factors.

			Univariate Analysis		Multivariate Analysis^a^		Multivariate Analysis^b^	
	Type	Type	Odds ratio		Adjusted odds ratio		Adjusted odds ratio	
Variable	I–III	IV–VII	(95% CI)	P value	(95% CI)	P value	(95% CI)	P value
Work time	8.51 (2.22)	7.97 (1.92)	0.90 (0.80–1.02)	0.09	0.97 (0.82–1.14)	0.69	0.95 (0.81–1.12)	0.58
Sleep time	7.18 (1.01)	6.78 (1.25)	0.69 (0.53–0.91)	7.74E-3	0.74 (0.52–1.05)	0.10	0.76 (0.54–1.09)	0.13
Sleep quality								
normal	168	25	1 [Reference]		1 [Reference]		1 [Reference]	
unsatisfactory	120	35	1.96 (1.12–3.45)	0.02	1.71 (0.80–3.64)	0.16	1.69 (0.79–3.63)	0.18
Sleep hours								
sufficient	275	50	1 [Reference]		1 [Reference]		1 [Reference]	
insufficient	13	10	4.23 (1.76–10.18)	1.28E-3	4.47 (1.07–18.68)	0.04	4.30 (1.02–18.18)	0.04
Ability to fall asleep								
normal	203	48	1 [Reference]		1 [Reference]		1 [Reference]	
difficult	85	23	1.49 (0.83–2.65)	0.18	2.06 (0.94–4.52)	0.71	2.10 (0.94–4.67)	0.07
Sleep disruption								
normal	241	48	1 [Reference]		1 [Reference]		1 [Reference]	
unsatisfactory	47	12	1.28 (0.63–2.60)	0.49	0.95 (0.35–2.60)	0.92	0.86 (0.30–2.45)	0.78
Scalp status								
normal	140	16	1 [Reference]		1 [Reference]		1 [Reference]	
abnormal	150	43	2.51 (1.35–4.66)	3.56E-3	2.57 (1.14–5.78)	0.02	3.02 (1.30–7.06)	0.01
Itchy scalp								
no	234	44	1 [Reference]		1 [Reference]		1 [Reference]	
yes	56	15	1.43 (0.74–2.74)	0.29	0.76 (0.28–2.04)	0.58	0.80 (0.30–2.19)	0.67
Oily scalp								
no	196	32	1 [Reference]		1 [Reference]		1 [Reference]	
yes	94	27	1.76 (1.00–3.10)	0.05	4.12 (1.71–9.97)	1.67E-3	4.65 (1.86–11.64)	1.67E-3
Hair dyeing								
no	251	54	1 [Reference]		1 [Reference]		1 [Reference]	
yes	42	6	0.66 (0.27–1.64)	0.38	0.58 (0.18–1.87)	0.36	0.69 (0.21–2.23)	0.53
Hair perming								
no	283	55	1 [Reference]		1 [Reference]		1 [Reference]	
yes	9	3	1.72 (0.45–6.54)	0.43	0.54 (0.04–6.51)	0.63	0.39 (0.03–5.12)	0.48
Smoking								
no	119	25	1 [Reference]		1 [Reference]		1 [Reference]	
yes	175	35	0.95 (0.54–1.67)	0.86	0.98 (0.46–2.08)	0.95	1.13 (0.52–2.44)	0.76
Alcohol consumption								
no	177	33	1 [Reference]		1 [Reference]		1 [Reference]	
yes	113	27	1.28 (0.73–2.25)	0.39	1.11 (0.52–2.34)	0.79	1.30(0.60–2.81)	0.51
Betel nut chewing								
no	215	39	1 [Reference]		1 [Reference]		1 [Reference]	
yes	79	21	1.47 (0.81–2.64)	0.20	1.18 (0.54–2.60)	0.68	1.27 (0.57–2.85)	0.56
Soy bean drink consumption								
never or seldom	206	50	1 [Reference]		1 [Reference]		1 [Reference]	
1–3 days or more per week	88	10	0.47 (0.23–0.97)	0.04	0.49 (0.19–1.26)	0.14	0.46 (0.17–0.12)	0.12
Coffee consumption								
never or seldom	201	43	1 [Reference]		1 [Reference]		1 [Reference]	
1–3 days or more per week	88	15	0.80 (0.42–1.51)	0.49	1.25 (0.53–2.93)	0.62	1.20 (0.51–2.87)	0.67
Dairy milk drink consumption								
never or seldom	200	44	1 [Reference]		1 [Reference]		1 [Reference]	
1–3 days or more per week	89	14	0.72 (0.37–1.37)	0.31	0.55 (0.23–1.32)	0.18	0.46 (0.19–1.15)	0.09
Cheese consumption								
never or seldom	268	57	1 [Reference]		1 [Reference]		1 [Reference]	
1–3 days or more per week	23	2	0.41 (0.09–1.78)	0.23	0.52 (0.10–2.69)	0.44	0.50 (0.09–2.64)	0.41

*Statistical significance with Bonferroni Correction (P<1.14E-3).

^a^ Adjusted by age and family history of AGA.

^b^ Adjusted by age, family history of AGA and residence area.

After adjusting for age and family history, the odds ratios for sleep deficiency (fewer than 6 hours per day) and scalp abnormalities were 4.47 (P = 0.04) and 2.57 (P = 0.02), respectively. In the multivariate analysis, after adjusting for age, family history and area of residence, the odds ratios for sleep deficiency and scalp abnormalities were 4.30 (P = 0.04) and 3.02 (P = 0.01), respectively.

### SNPs and AGA

The SNP analysis was conducted in 184 individuals who were randomly selected from the original pool of 354 subjects and their age-, residence area- and smoking status-matched controls (subjects exhibited moderate to severe AGA, and controls had no or only slight AGA). The matched variables sufficiently increased the statistical power with an efficient sample size.

Five loci (rs1998076, rs913063, rs1160312, rs201571 and rs11575897) were evaluated in the SNP genotyping tests. Primers for rs11575897 were generated using “Assay by Design” based on the two 500-bp fragments flanking the SNP. Unfortunately, these primers yielded poor results due to secondary structure formation or high GC content and failed to satisfy our quality control criteria; therefore, this SNP was excluded from the study. The genotype and allele frequencies of rs1998076, rs913063, rs1160312 and rs201571 in the two groups (AGA group and the control group) were in line with Hardy-Weinberg equilibrium (Table 4).

**Table 4:** Male AGA-associated genotypes and risk allele frequencies.

Marker	Chr.		Type I–III	Type IV–VII	Odds ratio (95% CI)	P value^a^	P value^b^
rs1998076	20	Genotype (n)	123	55		1.25E-2	0.10
		GG	39	30	1 [Reference]		
		AG	70	22	0.41 (0.21–0.80)	1.12E-2	
		AA	14	3	0.28 (0.07–1.06)	0.06	
		Dominant model					
		GG	39	30	1 [Reference]		
		AG+AA	84	25	0.39 (0.20–0.74)	5.31E-3	
		Recessive model					
		GG+AG	119	52	1 [Reference]		
		AA	14	3	0.45 (0.12–1.63)	0.22	
		Risk allele frequency			0.52 (0.31–0.85)	1.87E-3	
		G	0.60	0.75			
		A	0.40	0.25			
rs913063	20	Genotype	124	56		0.04	0.32
		CC	58	24	1 [Reference]		
		CA	57	17	0.72 (0.35–1.48)	0.37	
		AA	9	15	4.03 (1.55–10.45)	4.08E-3	
		Dominant model					
		CC	58	24	1 [Reference]		
		CA+AA	66	32	1.17 (0.62–2.21)	0.63	
		Recessive model					
		CC+CA	115	41	1 [Reference]		
		AA	9	15	4.68 (1.90–11.50)	7.44E-4*	
		Risk allele frequency			1.67 (1.05–2.65)	0.03	
		C	0.70	0.58			
		A	0.30	0.42			
rs1160312	20	Genotype	126	57		0.03	0.19
		GG	59	24	1 [Reference]		
		AG	57	17	0.73 (0.36–1.51)	0.40	
		AA	10	16	3.93 (1.57–9.89)	4.58E-3	
		Dominant model					
		GG	59	24	1 [Reference]		
		AG+AA	67	33	1.21 (0.64–2.28)	0.55	
		Recessive model					
		GG+AG	116	41	1 [Reference]		
		AA	10	16	4.53 (1.90–10.77)	8.75E-4*	
		Risk allele frequency			1.71 (1.08–2.71)	1.71E-2	
		G	0.69	0.57			
		A	0.31	0.43			
rs201571	20	Genotype	123	55		9.85E-3*	0.06
		CC	61	23	1 [Reference]		
		CT	52	16	0.82 (0.39–1.71)	0.59	
		TT	10	16	4.24 (1.68–10.69)	2.19E-3	
		Dominant model					
		CC	61	23	1 [Reference]		
		CT+TT	62	32	1.37 (0.72–2.60)	0.34	
		Recessive model					
		CC+CT	113	39	1 [Reference]		
		TT	10	16	4.64 (1.94–11.06)	4.87E-4*	
		Risk allele frequency			1.87 (1.17–2.98)	2.49E-3	
		C	0.71	0.56			
		T	0.29	0.44			

Statistical significance with Bonferroni Correction (P<1.14E-2).

^a^ The correlation analysis between AGA and genotype and between AGA and risk allele frequency was performed using logistic regression and chi-square test.

^b^ Verification of Hardy-Weinberg equilibrium.

The rs1998076 locus is located at position 21828045 on chromosome 20 in an intergenic region. The wild-type allele is homozygous GG, while the mutant allele is homozygous AA. The GG genotype was considered to be the control. We found odds ratios of 0.28 (P = 0.06) for the AA genotype and 0.41 (P<0.01) for the AG (heterozygous) genotype, respectively, for developing moderate to severe AGA. In the dominant model, the GG genotype was used as the control, and the combined odds ratio of the AA and AG genotypes was 0.39 (P<0.01) for developing moderate to severe AGA. In the recessive model, the GG and GA genotypes were used as the control, and the odds ratio of the AA genotype was 0.45 (P<0.01) for developing moderate to severe AGA. Compared with the G allele, the A allele had an odds ratio of 0.52 (p<0.01) for developing moderate to severe AGA.

The rs913063 locus is located at position 21990418 of chromosome 20, upstream of the non-coding gene RP11-125P18.1. The wild-type genotype is CC, and the mutant genotype is AA. Using CC as the control group, we found that the AA and CA genotypes had odds ratios of 4.03 (P<0.01) and 0.72 (P = 0.37), respectively, for developing moderate to severe AGA. In the dominant model, the CC genotype was used as the control, and the combined odds ratio of the CA and AA genotypes was 1.17 (P = 0.63) for developing moderate to severe AGA. In the recessive model, the CC and CA genotypes were used as the controls, and the AA genotype was found to have an odds ratio of 4.68 (P<0.01) for developing moderate to severe AGA. Altogether, compared to the C allele, the A allele carried an odds ratio of 1.67 (P = 0.03) for developing moderate to severe AGA.

The rs1160312 locus is located at position 21998503 of chromosome 20, inside the non-coding gene RP11-125P18.1. The wild-type genotype is GG, while the mutant genotype is AA. Using the GG genotype as the control, we found that the AA and AG genotypes carried odds ratios of 3.93 (P<0.01) and 0.73 (P = 0.40), respectively, for developing moderate to severe AGA. In the dominant model, the GG genotype was used as the control, and the combined odds ratio of the AA and AG genotypes was 1.21 (P = 0.55) for developing moderate to severe AGA. In the recessive model, the GG and AG genotypes were used as the controls, and AA was found to harbor an odds ratio of 4.53 (P<0.01) for developing moderate to severe AGA. Hence, compared with the G allele, the A allele had an odds ratio of 1.71 (P = 0.02) for developing moderate to severe AGA.

The rs201571 locus is located at position 21961514 of chromosome 20, within an intergenic region. The wild-type genotype is CC, while the mutant genotype is TT. Using the CC genotype as the control, we found that the TT and CT genotypes harbored odds ratios of 4.24 (P<0.01) and 0.82 (P = 0.59), respectively, for developing moderate to severe AGA. In the recessive model, the CC and CT genotypes were used as the control, and TT was found to have an odds ratio of 1.87 (P<0.01) for developing moderate to severe AGA. Therefore, compared with the C allele, the T allele had an odds ratio of 1.87 (p<0.01) for developing moderate to severe AGA.

### AGA and models of the related factors

In addition to comparing the genotypes, residence area and smoking status, other factors with significant odds ratios for the development of moderate to severe AGA were subjected to logistic regression analysis (Table 5). These factors included blood concentrations of vanadium, the AA genotype of rs1160312, sleep deficiency (fewer than 6 hours per day) and frequent consumption of soy bean drinks (3 days per week), which had odds ratios (represented here with their respective 95% confidence intervals) of 17.67 (5.48–57.00), 0.981 (0.972–0.991), 10.75 (3.12–37.03), 6.99 (1.29–37.87) and 0.23 (0.08–0.69), respectively, for moderate to severe AGA. The Hosmer-Lemeshow test revealed that the goodness of fit was not statistically significant (P = 0.50). The explained variance (Nagelkerke R^2^) was 0.64, the accuracy was 0.861 and the AUC was 0.926 (95% CI: 0.839–0.962). The interactions between the SNPs and the environmental or personal behavioral risk factors for AGA were not statistically significant in these models.

**Table 5:** The optimal model of male AGA and related factors.

Variables			Multivariate Analysis^a^		Multivariate Analysis^b^	
	Type	Type	Adjusted odds ratio		Adjusted odds ratio	
	I–III	IV–VII	(95% CI)	P value	(95% CI)	P value
rs1160312						
GG+AG	59+57	24+17	1 [Reference]		1 [Reference]	
AA	10	16	7.30 (2.76–10.08)	7.34E-5*	7.65 (2.54–11.53)	3.05E-4*
Blood vanadium	117.49	93.33	0.987 (0.981–0.993)	2.70E-4*	0.985 (0.978–0.992)	1.14E-3*
Sleep hours						
sufficient	275	50			1 [Reference]	
deficient	13	10			3.45 (1.08–11.03)	3.65E-3*
Soy bean drink consumption						
never or seldom	206	50	-		1 [Reference]	
1–3 days or more per week	88	10	-	-	0.38 (0.15–0.78)	4.30E-3*
Scalp status						
normal	140	16			1 [Reference]	
abnormal	150	43			2.29 (2.76–10.08)	3.27E-3

Statistical significance with Bonferroni Correction (P<1.14E-3).

^a^ The model was applied to rs1160312 and blood vanadium.

^b^ The model was applied to rs116031, blood vanadium, sleep deficiency (fewer than 6 hours per day), soy bean drink consumption and scalp status. CI: Odds ratio confidence interval. Blood vanadium concentration (per unit), sufficient sleep hours and frequent soy bean drink consumption are associated with approximately 2%, 86% and 77% decreases in the risk of AGA, respectively.

## Discussion

This study found that the prevalence of moderate to severe AGA in men in our study areas is 17% (60/354), higher than previously reported [14]. The prevalence of AGA in men increased with age. Age, family history, poor sleep quality, scalp abnormalities (itchy, oily or dry), and drinking soy bean products regularly were still important factors for moderate to severe AGA. The body concentrations of all tested heavy metals and the genotypes of the rs1998076, rs913063, rs1160312 and rs201571 polymorphisms were all correlated to the development of moderate to severe AGA.

### AGA and physiological concentrations of heavy metals

In 1978, vanadium was found to be an essential trace element in humans and animals [27]. The necessity of vanadium has been confirmed by the World Health Organization [28]. Most often found in seafood, vanadium is involved in a variety of biological processes, including hematopoiesis, the maintenance of blood pressure, growth promotion, the maintenance of cholesterol levels, and the stimulation of receptors and other enzymes that phosphorylate insulin and regulate its biological activity. In sugar metabolism, vanadium mainly facilitates the entry of glucose into cells as a hypoglycemic agent. Vanadium deficiency can lead to many characteristics, including increased cholesterol, anemia, myocardial weakness and diabetes [28]. Furthermore, studies have shown that hypertension, which is itself closely associated with high cholesterol, and impaired glucose tolerance (or Type II diabetes) are both associated with the development of AGA [4],[29]. Therefore, the role of vanadium in humans requires further investigation. Vanadium deficiency in poultry has been shown to cause incomplete feather coverage or apparent slower feather growth [30].

Interestingly, there is a significant discrepancy between this study and one previously performed by Naginiene *et al.* [18], who reported that the hair, blood and urine of bald individuals contain increased concentrations of lead, copper and cadmium but decreased concentrations of zinc. Our data showed that individuals with moderate to severe AGA had decreased concentrations of lead, copper, cadmium and zinc in their blood and increased concentrations of lead, cadmium and zinc in their urine (the copper concentration was similar to that of the control). One possible cause for these differences is the different racial backgrounds of the subjects in the two studies. Additionally, the sampling methods were different; Naginiene *et al.* [18] used children as the control group and recruited adult men and women for the bald group, while this study used adult men with and without AGA in the bald and control groups. Due to these methodological differences, it is difficult to directly compare the two studies. Lastly, we found a significant correlation between blood vanadium concentration and protection against moderate to severe AGA that has not been reported previously; Naginiene *et al.* did not examine vanadium concentrations.

### Dietary factors, environmental factors, personal behaviors and AGA

Although Su and Chen found an association between smoking and AGA [14], our results are consistent with other previous studies [31], [32], [33] that failed to reveal a significant correlation between cigarette smoking and AGA. This inconsistency may be caused by differences in the sampling methods and subject demographics. The present study did not exclude smoking as a risk factor, and the measurements included a detailed record of smoking frequency per day and age of smoking commencement. Nevertheless, to avoid complications based on smoking, we balanced the ratios of smokers and non-smokers in both the control and AGA groups in this study.

Our data on environmental factors, personal behaviors and dietary intake indicated that sleep deprivation, oily scalp and the frequent consumption of soy bean drinks are correlated with moderate to severe AGA. A previous study revealed that insufficient sleep is a risk factor for sebaceous gland diseases, including seborrheic dermatitis, acne, AGA and rosacea [34]. Oily scalp as a risk factor for moderate to severe AGA can be explained as follows: Oily skin results from robust secretion from the sebaceous glands, which are controlled by androgens. This increased secretion in turn causes changes to the hair cycle and subsequently aggravates the manifestation of AGA [35], [36], [37]. Our data suggest that sleeping for fewer than 6 hours each day increases the risk of AGA. However, because this was a case-control study, we were unable to deduce a cause and effect relationship or identify the specific mechanisms underlying this correlation. Further investigation is needed to address these issues.

We found that frequent soy bean drink consumption is protective against moderate to severe AGA. Soy bean drinks are rich in isoflavones, and the isoflavone metabolite equol displays high levels of antioxidant [38], [39], [40] and estrogen-like activities [38], [41], [42], [43]. There are many reports that isoflavone [44], [45], antioxidants [46] and estrogens [43], [47], [48], [49], [50], [51], [52], [53], [54], [55], [56], [57], [58] are protective against alopecia. The previous reports found that orally administered soymetide-4 (MITL), a soy-derived immunostimulating peptide from soy bean beta-conglycinin alpha' subunit, suppressed the alopecia induced by the anti-cancer drug etoposide [52], [59], [60], [61], [62]. Hypothetically, soy oil compounds may act to modify alopecia susceptibility by modulating estrogen-dependent mechanisms or inflammatory activity. Further studies are needed to explore the connections between isoflavone, equol and AGA.

### Genes and AGA

Regarding the relationship between chromosome 20 and AGA, this report is consistent with other relevant studies [8], [10]. Hillmer *et al.* showed that the risk-associated SNP rs2180439[T] is in linkage disequilibrium with rs1998076[G]. This is consistent with our findings that rs1998076[A] is a protective SNP, while rs202571[T] is a risk-associated SNP. The study by Richards *et al.* revealed that the r^2^ between rs1160312 and rs913063 is 1, indicating complete linkage disequilibrium. In other words, the information concerning rs913063 can be obtained by examining rs1160312. Although this linkage was reported previously, we performed genotyping to confirm this hypothesis. We found that both rs1160312[A] and rs913063[A] are risk-associated SNPs, corroborating the previous studies by Hillmer *et al.* and Richards *et al.* [8], [10]

Both rs1998076 and rs201571 are located in intergenic regions, while both rs1160312 and rs913063 are within the non-coding gene RP11-125P18.1. No transcription has been reported in any of these regions. However, previous studies have suggested that these SNPs might interact with *PAX1* [8], [10], which is highly expressed in the scalp. Although *PAX1* is outside of the LD region, its expression pattern indicates that it affects AGA, possibly due to altering the expression of other loci in this LD region.

It has been reported that the *AR* gene on the X chromosome is strongly associated with male AGA [11], [22], [23]. However, there are no polymorphisms in this gene among Asian populations and also in rs6152 of the present study. In addition, there are two SNPs within *SRY* [16]. The lack of AGA-associated SNP polymorphisms among Asian populations may underlie the lower frequency of AGA in this ethnic group. Sehgal *et al.* reported that a gradual shift in the type of AGA from the earlier types (II and III) to more severe types (VI) occurs significantly with increasing age [63]. Pathomvanich *et al.* reported that the prevalence of AGA Norwood III–VII was 38.52% and significantly increased with age in a study of 1124 men [64]. The lack of polymorphisms in AGA-associated SNPs among Asians might suggest a role for DNA methylation which results in changes in gene expression. This could explain why age is correlated with the prevalence of AGA [63], [64], [65], [66]. Further studies are needed to explore the connection between the lack of polymorphisms, DNA methylation and AGA among Asians. Further studies are also needed to explore the connection between the lacks of polymorphisms, DNA methylation and AGA.

Alopecia is a complex skin disorder observed in individuals whose conscious experience of distress is often absent and may be precipitated by environmental events, not simply the influence of inherited factors [67]. The contribution of genetic factors to alopecia is strong, but environmental factors, such as environmental stress, still play an important role, and the genetics of alopecia are consistent with a polygenic additive mode of inheritance [68]. This study examined whether any evidence for an environmental component to the risk for AGA exists; our results indicated that the genes and environmental factors studied account for a proportion of the risk of AGA. One limitation of this study is that AGA may be affected by many genes that have not yet been identified; twin studies have shown that the AGA condition is heritable, and a family history of AGA has been included in our analysis for this reason. However, there are still many findings [69], [70], [71], [72], [73], [74], [75], [76], [77], [78], [79], [80], [81], [82], [83], [84], [85], [86], [87], [88] that suggest that heritable and epigenetic [69], [89] differences also play a role in alopecia; this may replace the classical discussion of the roles of genetic and environmental factors in alopecia.

The environmental factors examined have been adequately reported, and the basis and the mechanistic explanations provided were congruent with previous findings [67], [68], [69], [72], [89], [90], [91], [92], [93], [94]. The cross-sectional nature of the measurements of blood metals and personal behaviors (for example, sleep patterns [67], [68], [70], [94] and oily scalp [62], [95], [96], [97], [98], [99], [100]) limit our ability to identify causal relationships; the time of onset of moderate to severe hair loss will need more investigation in future work.

Finally, we made an effort to adjust our independent variables in multiple logistic regression analyses. This adjustment identified several significant factors. Additional research will be required to investigate the effect of epigenetic changes on alopecia. Our epidemiological analyses are based on a sample that is representative of the Taiwanese population. Cases and controls were defined by AGA level, which was evaluated based on the Hamilton-Norwood scale by dermatological specialists at the Public Health Centers. The use of the Hamilton-Norwood scale ensured that baldness was accurately assessed according to international criteria [31], [101], [102], [103], [104], [105], [106], [107].

### The optimal model

It has been reported that genes only contribute to 13.7% of the explained variance in AGA [8]. Thus, in this study, family history, the concentration of vanadium in blood, the AA genotype of rs1160312 and the regular consumption of soy bean drinks (3 days per week) were examined in a logistic regression analysis. The results showed that these factors contributed to 59% of the explained variance of AGA. Furthermore, using ROC curve analysis, we found that rs1160312 is unable to completely account for all AGA-associated genetic factors and thus cannot replace the influence of family history on AGA risk.

### Conclusion

This study identified several AGA-associated factors, including the consumption of soy bean drinks, blood concentration of vanadium, the AA genotype of rs1160312, sleep patterns and scalp status. Blood vanadium and frequent consumption of soy bean drinks may provide protective effects.

## Supporting Information

File S1 **Supporting tables.** Table S1. SNP loci reported to be associated with AGA. Table S2. AGA-associated SNP loci investigated in this study. Table S3. Genotype details.
(DOCX)
