# Correction: Androgenic Alopecia Is Associated with Less Dietary Soy, Higher Blood Vanadium and rs1160312 1 Polymorphism in Taiwanese Communities

**DOI:** 10.1371/journal.pone.0105915

**Published:** 2014-08-11

**Authors:** The PLOS ONE Staff

In the title of this article, the word “Higher” should be “Lower.” The correct title is: Androgenic Alopecia Is Associated with Less Dietary Soy, Lower Blood Vanadium and rs1160312 1 Polymorphism in Taiwanese Communities. The correct citation is: Lai C-H, Chu N-F, Chang C-W, Wang S-L, Yang H-C, et al. (2013) Androgenic Alopecia Is Associated with Less Dietary Soy, Lower Blood Vanadium and rs1160312 1 Polymorphism in Taiwanese Communities. PLoS ONE 8(12): e79789. doi:10.1371/journal.pone.0079789
