# Supplementary material for: Androgenic Alopecia Is Associated with Less Dietary Soy, Higher Blood Vanadium and rs1160312 1 Polymorphism in Taiwanese Communities
Source: PLoS One. 2013 Dec 30;8(12):e79789. doi: 10.1371/journal.pone.0079789 (PMC3875420; doi:10.1371/journal.pone.0079789)

Table S1 SNP loci reported to be associated with AGA

| SNP | locus | OR | 95% CI | P value | author | country | year/month |
| --- | --- | --- | --- | --- | --- | --- | --- |
| rs925391 | Xq12-Xq13 (66,256,990-66,257,335 bp) | 11.15 | 4.65–27.42 | 1.98×10^-10^ | Hillmer et al. | Germany | 2005/Mar |
| rs962458 | Xq12-Xq13 (66,662,543-66,662,835 bp) | 11.1 | 3.27–37.74 | 2.56×10^-6^ |  |  |  |
| rs1337080 | Xq12-Xq13 (66,795,614-66,795,674 bp) | 10.78 | 3.12–36.19 | 4.75×10^-6^ |  |  |  |
| rs2207081 | Xq12-Xq13 (66,490,367-66,490,479 bp) | 10.72 | 4.89–23.75 | 5.04×10^-12^ |  |  |  |
| rs10521339 | Xq12-Xq13 (66,424,740-66,425,480 bp) | 9.89 | 4.62–20.7 | 2.1×10^-12^ |  |  |  |
| rs5919287 | Xq12-Xq13 (66,277,070-66,277,610 bp) | 9.7 | 4.22–22.45 | 1.76×10^-10^ |  |  |  |
| rs938059 | Xq12-Xq13 (66,253K-66,253,900 bp) | 9.63 | 4.18–22.17 | 9.2×10^-10^ |  |  |  |
| rs532649 | Xq12-Xq13 (66,183,240-66,184,710 bp) | 9.62 | 4.17–22.13 | 2.33×10^-10^ |  |  |  |
| rs989345 | Xq12-Xq13 (66,217,658-66,217,860 bp) | 9.56 | 4.2–22.29 | 6.13×10^-10^ |  |  |  |
| rs2223841 | Xq12-Xq13 (66,486,230-66,487,560 bp) | 9.48 | 4.48–20.05 | 1.72×10^-11^ |  |  |  |
| rs1041668 | Xq12-Xq13 (66,002,240-66,003,120 bp) | 9.29 | 4.03–21.44 | 1.58×10^-9^ |  |  |  |
| rs2221799 | Xq12-Xq13 (66,293,120-66,293,560 bp) | 9.21 | 4.04–21.47 | 1.39×10^-9^ |  |  |  |
| rs2497935 | Xq12-Xq13 (66,580,932-66,581,050 bp) | 8.69 | 4.23–17.87 | 3.17×10^-11^ |  |  |  |
| rs6152 | Xq12-Xq13 (66,681,880-66,682,820 bp) | 8.21 | 3.86–17.45 | 6.66×10^-10^ |  |  |  |
| rs5919393 | Xq12-Xq13 (66,741,790-66,742,380 bp) | 7.88 | 3.7–16.77 | 1.69×10^-8^ |  |  |  |
| rs1204038 | Xq12-Xq13 (66,704,730-66,705,170 bp) | 7.72 | 3.62–16.46 | 2.83×10^-9^ |  |  |  |
| rs5964577 | Xq12-Xq13 (66,088,036-66,088,396 bp) | 7.02 | 3.29–15.08 | 1.2×10^-8^ |  |  |  |
| rs1385695 | Xq12-Xq13 (65,820,610-65,821,860 bp) | 6.57 | 3.17–13.63 | 2.4×10^-8^ |  |  |  |
| rs775362 | Xq12-Xq13 (65,895,886-65,895,911 bp) | 6.4 | 3.11–13.39 | 3.73×10^-8^ |  |  |  |
| rs1158928 | Xq11-Xq12 (65,665,210-65,666,630 bp) | 3.22 | 1.85–5.61 | 2.08×10^-5^ |  |  |  |
| rs1485682 | Xq11-Xq12 (65,732,159-65,732,474 bp) | 3.16 | 1.81–5.48 | 3.08×10^-5^ |  |  |  |
| rs1385699 | Xq11-Xq12 (65,741,589-65,741,834 bp) | 3 | 1.71–5.32 | 9.41×10^-5^ |  |  |  |
| rs1157321 | Xq12-Xq13 (67,238,240-67,238,690 bp) | 2.93 | 1.75–4.87 | 2.73×10^-5^ |  |  |  |
| rs2781516 | Xq12-Xq13 (67,018,677-67,018,835 bp) | 2.69 | 1.56–4.64 | 2.86×10^-4^ |  |  |  |
| rs1379146 | Xq12-Xq13 (65,522,928-65,523,244 bp) | 2.6 | 1.51–4.5 | 4.72×10^4^ |  |  |  |
| rs2885913 | Xq12-Xq13 (67,032,330-67,032,730 bp) | 2.53 | 1.62–3.97 | 4.29×10^-5^ |  |  |  |
| rs2363785 | Xq12-Xq13 (67,087,865-67,088,181 bp) | 2.34 | 1.49–3.68 | 2.04×10^-4^ |  |  |  |
| rs708969 | Xq11-Xq12 (65,386,462-65,386,778 bp) | 2.27 | 1.29–4 | 3.9×10^-3^ |  |  |  |
| rs1936313 | Xq12-Xq13 (67,117,269-67,117,477 bp) | 2.15 | 1.36–3.37 | 9.15×10^-4^ |  |  |  |
| rs1410127 | Xq12-Xq13 (67,196,948-67,197,264 bp) | 1.71 | 1.08–2.67 | 2.13×10^-2^ |  |  |  |
| rs492933 | Xq12-Xq13 (67,180,503-67,180,635 bp) | 1.69 | 1.07–2.65 | 2.39×10^-2^ |  |  |  |
| rs6152 | Xq12-Xq13 (66,681,880-66,682,820 bp) | 0.54 | (0.36-0.81) | 0.003 | Ellis et al. | Australia | 2007/Jan |
| rs6625163[A] | Xq12-Xq13 (66,427,645-66,427,773 bp) | 3.30 | (2.31–4.71) | 5.0×10^-11^ | Richards et al. | London | 2008/Nov |
| rs913063[T] | 20p11.1-20p11.2 (21,990,337-21,990,499 bp) | 1.80 | (1.49–2.16) | 3.5×10^-10^ |  |  |  |
| rs1160312[A] | 20p11.1-20p11.2 (21,998,422-21,998,584 bp) | 1.79 | (1.49–2.15) | 3.2×10^-10^ |  |  |  |
| rs1998076 | 20p11.1-20p11.2 (21,827,989-21,828,101 bp) | 1.90 | (1.50-2.41) | 1.30×10^-7^ | Hillmer et al. | Germany | 2008/Nov |
| rs2180439 | 20p11.1-20p11.2 (21,800,984-21,801,216 bp) | 1.82 | (1.45-2.30) | 3.85×10^-7^ |  |  |  |
| rs6137444 | 20p11.1-20p11.2 (21,733,505-21,733,773 bp) | 1.74 | (1.37-2.21) | 3.11×10^-6^ |  |  |  |
| rs201571 | 20p11.1-20p11.2 (21,961,130-21,961,900 bp) | 1.72 | (1.36-2.17) | 4.31×10^-6^ |  |  |  |
| rs6113491 | 20p11.1-20p11.2 (22,005,230-22,005,600 bp) | 1.66 | (1.33-2.08) | 8.63×10^-6^ |  |  |  |
| rs2534636 | (SRY)_Y_2717176 | CACATAGGTGAACCTTGAAAATGTTA[C/T]  ACTGTGTGAAAAAGTCAGATACAAG | | | Chen et al. | Taiwan | 2003/Oct |
| rs11575897 | (SRY)_Y_2715180 | GCAGATCCCGCTTCGGTACTCTGCAG[C/T]  GAAGTGCAACTGGACAACAGGTTGT | | |  |  |  |

Table S2 AGA-associated SNP loci investigated in this study

| SNP | locus | OR | 95% CI | P value | author | country | year/month |
| --- | --- | --- | --- | --- | --- | --- | --- |
| rs925391 | Xq12-Xq13 (66,256,990-66,257,335 bp) | 11.15 | 4.65–27.42 | 1.98×10-10 | Hillmer et al. | Germany | 2005/Mar |
| rs10521339 | Xq12-Xq13 (66,424,740-66,425,480 bp) | 9.89 | 4.62–20.7 | 2.1×10-12 | Hillmer et al. | Germany | 2005/Mar |
| rs6625163[A] | Xq12-Xq13 (66,427,645-66,427,773 bp) | 3.3 | 2.31–4.71 | 5.0×10-11 | Richards et al. | London | 2008/Nov |
| rs1158928 | Xq11-Xq12 (65,665,210-65,666,630 bp) | 3.22 | 1.85–5.61 | 2.08×10-5 | Hillmer et al. | Germany | 2005/Mar |
| rs1998076 | 20p11.1-20p11.2 (21,827,989-21,828,101 bp) | 1.9 | 1.50-2.41 | 1.30×10-7 | Hillmer et al. | Germany | 2008/Nov |
| rs913063[T] | 20p11.1-20p11.2 (21,990,337-21,990,499 bp) | 1.8 | 1.49–2.16 | 3.5×10-10 | Richards et al. | London | 2008/Nov |
| rs1160312[A] | 20p11.1-20p11.2 (21,998,422-21,998,584 bp) | 1.79 | 1.49–2.15 | 3.2×10-10 | Richards et al. | London | 2008/Nov |
| rs201571 | 20p11.1-20p11.2 (21,961,130-21,961,900 bp) | 1.72 | 1.36-2.17 | 4.31×10-6 | Hillmer et al. | Germany | 2008/Nov |
| rs6152 | Xq12-Xq13 (66,681,880-66,682,820 bp) | 0.54 | 0.36-0.81 | 0.003 | Ellis et al. | Australia | 2007/Jan |
| rs2534636 | (SRY)_Y_2717176 | CACATAGGTGAACCTTGAAAATGTTA[C/T]  ACTGTGTGAAAAAGTCAGATACAAG | | | Chen et al. | Taiwan | 2003/Oct |
| rs11575897 | (SRY)_Y_2715180 | GCAGATCCCGCTTCGGTACTCTGCAG[C/T]  GAAGTGCAACTGGACAACAGGTTGT | | | Chen et al. | Taiwan | 2003/Oct |

Table S3 Genotype details


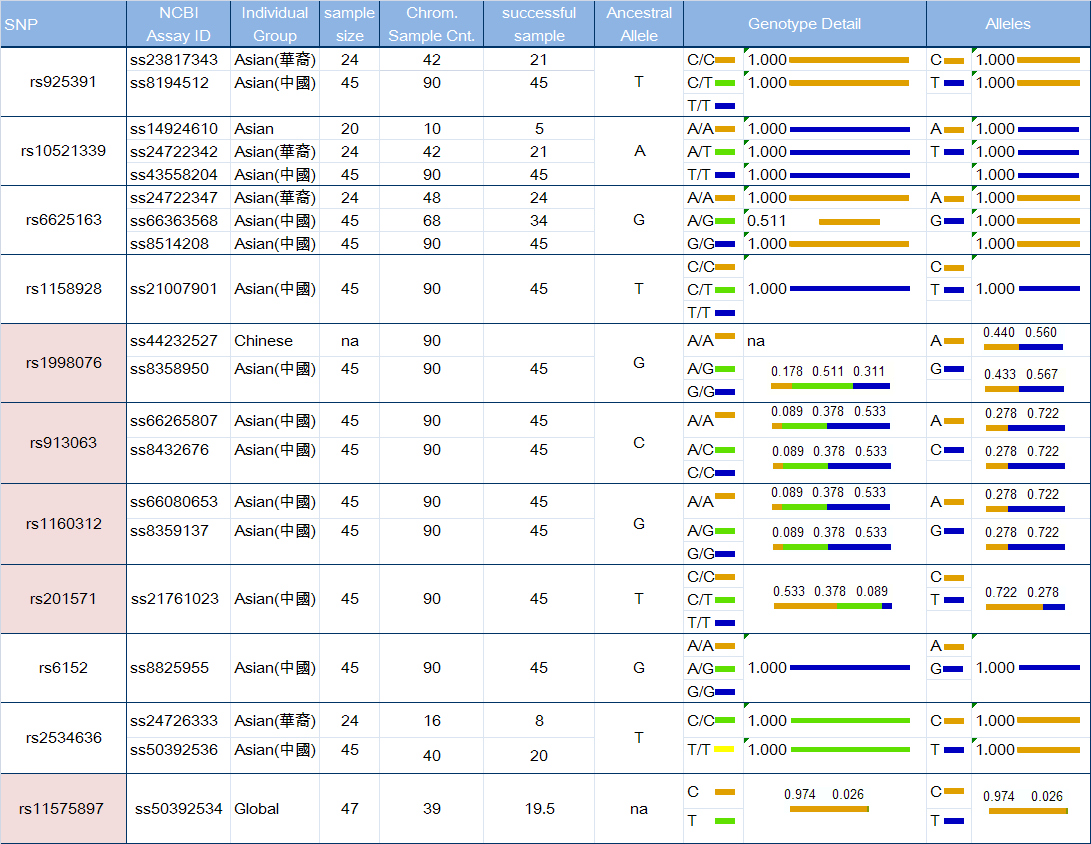

Supplement: File S1 — Supporting tables. Table S1. SNP loci reported to be associated with AGA. Table S2. AGA-associated SNP loci investigated in this study. Table S3. Genotype details. (DOCX) [file pone.0079789.s001.docx]
